# PARTNER CORRESPONDENCE IN SOCIAL INTERACTION TIME AND WELL-BEING: EVERYDAY EVIDENCE FROM OLDER DYADS

**DOI:** 10.1093/geroni/igae098.3216

**Published:** 2024-12-31

**Authors:** Carlotta Grünjes, Elizabeth Zambrano Garza, Rachel Murphy, Kenneth Madden, Denis Gerstorf, Gizem Hueluer, Christiane Hoppmann

**Affiliations:** University of Bonn, Bonn, Nordrhein-Westfalen, Germany; University of British Columbia, Vancouver, British Columbia, Canada; University of British Columbia, Vancouver, British Columbia, Canada; University of British Columbia, Vancouver, British Columbia, Canada; Humboldt University Berlin, Berlin, Berlin, Germany; University of Bonn, Bonn, Nordrhein-Westfalen, Germany; The University of British Columbia, Vancouver, British Columbia, Canada

## Abstract

Social interactions play an important role in older adults’ daily well-being. Longer duration of interaction has been associated with higher well-being at the individual level. However, less is known about the extent to which partners differ in their convergence in interaction reports and whether such differences might be associated with daily well-being. Positive Illusion theory states that individuals are more content in relationships if they idealize their partner leading to the assumption that overreporting interaction time may benefit well-being. Based on the literature, we expect that longer social interaction time is associated with higher well-being at the individual level. At the partner level, we look at discrepancies and similarities. We assess whether individuals report higher well-being on days when they overreport the interaction time with their partner. We further test whether partners who are more similar in evaluating the time spent together are also more similar in their well-being reports. This study uses dyadic end-of-day diary data from 136 Canadian older adults (M = 66.68 years, SD = 13.11 range: 18-87 years, 60% women) and a close other of their choice. Preliminary analyses indicate that longer duration of interaction was associated with higher well-being. Correlations show differences in interaction time reports between partners. Multilevel models will examine whether overreporting interaction time may be associated with well-being. Further analyses will investigate how synchrony in interaction time reports shape joint well-being. As data collection is completed, these results will be ready to be presented by the time of GSA conference in November.

